# Microbial community diversity patterns are related to physical and chemical differences among temperate lakes near Beaver Island, MI

**DOI:** 10.7717/peerj.3937

**Published:** 2017-10-16

**Authors:** Miranda H. Hengy, Dean J. Horton, Donald G. Uzarski, Deric R. Learman

**Affiliations:** Institute for Great Lakes Research and Department of Biology, Central Michigan University, Mount Pleasant, MI, United States of America

**Keywords:** Freshwater lakes, 16S rRNA, Microbial communities, Stratification

## Abstract

Lakes are dynamic and complex ecosystems that can be influenced by physical, chemical, and biological processes. Additionally, individual lakes are often chemically and physically distinct, even within the same geographic region. Here we show that differences in physicochemical conditions among freshwater lakes located on (and around) the same island, as well as within the water column of each lake, are significantly related to aquatic microbial community diversity. Water samples were collected over time from the surface and bottom-water within four freshwater lakes located around Beaver Island, MI within the Laurentian Great Lakes region. Three of the sampled lakes experienced seasonal lake mixing events, impacting either O_2_, pH, temperature, or a combination of the three. Microbial community alpha and beta diversity were assessed and individual microbial taxa were identified via high-throughput sequencing of the 16S rRNA gene. Results demonstrated that physical and chemical variability (temperature, dissolved oxygen, and pH) were significantly related to divergence in the beta diversity of surface and bottom-water microbial communities. Despite its correlation to microbial community structure in unconstrained analyses, constrained analyses demonstrated that dissolved organic carbon (DOC) concentration was not strongly related to microbial community structure among or within lakes. Additionally, several taxa were correlated (either positively or negatively) to environmental variables, which could be related to aerobic and anaerobic metabolisms. This study highlights the measurable relationships between environmental conditions and microbial communities within freshwater temperate lakes around the same island.

## Introduction

Lakes are complex ecosystems that span a range of physical and chemical properties, which are driven by differences in formation, hydrology, weather patterns, and geology (Wetzel, 2001). Further, even lakes within the same geographic region can vary widely in physicochemical conditions, both spatially and temporally based on formation, age, and trophic status (Clement, Murry & Uzarski, 2015). The physical and chemical attributes of a lake can impact microbial communities and the biogeochemical processes they mediate, since microbial communities are governed by local environmental conditions. The essential processes regulated by microbial communities include, but are not limited to, nutrient cycling (e.g., carbon, nitrogen, and sulfur), which supports biologically suitable environmental conditions within lakes (Essington & Carpenter, 2000), as well as chemical export, such as respiration of CO_2_, and other redox-sensitive elements (Paerl & Pinckney, 1996; Pilcher et al., 2015). As microbial community function is related to microbial community composition (Bier et al., 2015), and community composition is constrained by local environmental conditions, it is important to explore microbial communities within individual lakes.

While environmental conditions are unique to each lake, environmental gradients can also occur within some lakes that physicochemically stratify. Water column mixing, or turnover, followed by a return to stratified conditions is a natural ecosystem disturbance that occurs seasonally in many lakes. This phenomenon is known to influence microbial communities, as a consequence of shifting environmental conditions, and even impacts microbial community assembly mechanisms (Tammert, Kisand & Nõges, 2005; Shade, Jones & McMahon, 2008;  Shade, Chiu & McMahon, 2010a;  Shade, Chiu & McMahon, 2010b; Shade et al., 2011; Shade et al., 2012b; Garcia et al., 2013; Meuser et al., 2013; Andrei et al., 2015). The stratification of water masses at different temperatures and densities results in a hypolimnion that is not only colder, but tends to have lower dissolved oxygen and pH relative to the epilimnion as the rate of decomposition tends to exceed photosynthesis (Fenchel & Finlay, 2008). Furthermore, inorganic nutrients (e.g., C, N, and P) may accumulate in the hypolimnion (Tõnno, Ott & Nõges, 2005; Zadereev, Tolomeev & Drobotov, 2014). Lake mixing events can transport dissolved organic carbon (DOC; described as the amount of C within a system) throughout lakes (Mostofa et al., 2005; Kim, Nishimura & Nagata, 2006; Li et al., 2008), and dissolved organic matter (DOM; quality of organic matter as described in Chappaz & Curtis, 2013) has previously been shown to vary between upper and lower layers of lakes (Mostofa et al., 2005). This suggests that structurally different organic compounds may not only differ among lakes, but also characterize each layer in some lakes. In addition, both DOC and DOM have been found to shape microbial community composition depending upon carbon source and concentration (Cotner & Biddanda, 2002; Burkert et al., 2003; Crump et al., 2003; Eiler et al., 2003; Grossart et al., 2008; Amaral, Graeber & Calliari, 2016; Lucas et al., 2016). As previously stated, chemical and physical components are major drivers of bacterial community structure and population shifts, therefore, lake stratification can present a major disturbance for bacterial communities and may impact microbial communities structure as lakes gradually stratify post-mixing.

Research to date demonstrates that microbial communities respond to disturbance with various degrees of resistance and resilience, depending upon the existing community and qualities of the disturbance (Allison & Martiny, 2008; Shade et al., 2011). For example, microbial communities may show resistance to lake mixing and physicochemical stratification, remaining unaffected in the face of disturbance (Shade et al., 2012a). However, depending upon the physicochemical attributes disturbed (e.g., O_2_, nutrients, pH, specific conductance etc.), disturbance influences microbial communities differentially in extent of community change, resistance, and resilience (Shade et al., 2011). Additionally, different subsets of microbes within a community (e.g., generalist vs. rare taxa) can experience different patterns of resistance and resilience. Illustrating this, Shade, Chiu & McMahon (2010b) found that many generalist taxa are resistant to mixing and subsequent changes of temperature and dissolved oxygen levels. Nevertheless, individual taxa (often specialist or rare) can be positively or negatively influenced as a result of physicochemical shifts and show fundamentally different reactions to mixing than dominant community members (Shade, Chiu & McMahon, 2010a; Shade, Chiu & McMahon, 2010b). As such, microbial communities can vary between lakes due to differences in lake chemistry, as well as within lakes at finer scales for the same reason.

In this study, three freshwater inland lakes of Beaver Island, Michigan, USA, as well as an adjacent location within Lake Michigan, were sampled to evaluate the relationship between microbial communities and local physicochemistry within surface-water and bottom-water habitats (epilimnion and hypolimnion during stratification). These lakes were selected as they each hosted unique and contrasting physicochemical properties (Clement, Murry & Uzarski 2015). Two of the lakes were holomictic and experienced oxygen stratification, while another holomictic lake (Lake Michigan) did not experience stratification at the point of sampling, but did experience a thermocline. The final lake (Barney’s Lake) is a shallow lake which did not experience a mixing event and lacked physicochemically stratified layers. Specifically, we sought to explore the relationships between microbial community diversity and environmental variables known to stratify within lakes. We also explored microbial community diversity change over time within each lake with respect to post-mixing stratification of environmental variables or a lack thereof. Physical and chemical parameters were collected in conjunction with high resolution microbial community data (via 16S rRNA gene sequencing) to explore relationships between microbial taxa and natural physicochemical gradients among and within sampled lake systems.

## Methods

### Sampling locations

**Figure 1:**
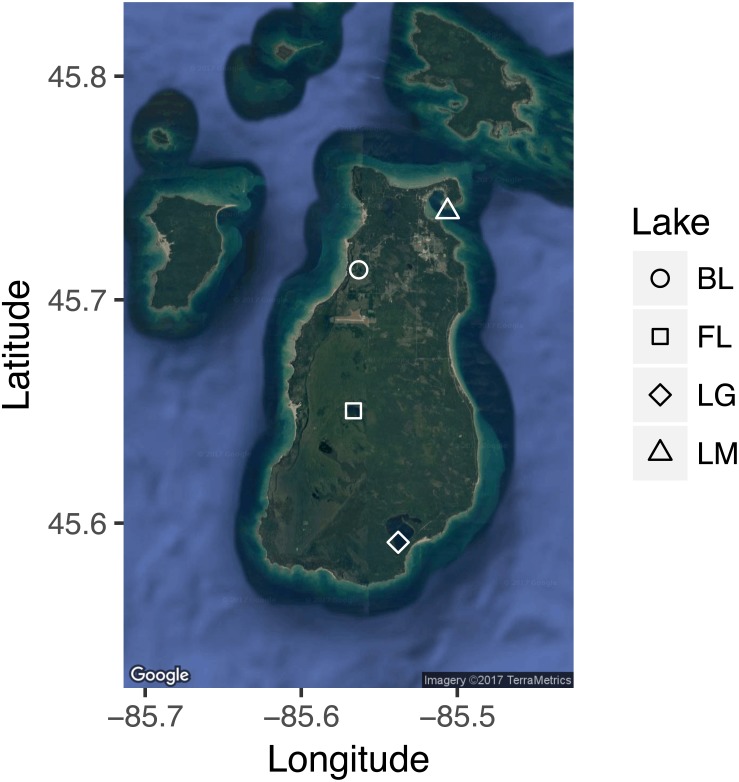
Map of sampling region. Map of sampled lakes in the Beaver Island region. Shapes correspond to lake sampled. (Map data^©^ 2017 Google; 2017 TerraMetrics, Inc., http://www.terrametrics.com).

Three inland lakes on Beaver Island, MI (Fox Lake [FL], Barney’s Lake [BL], and Lake Geneserath [LG], located on Beaver Island, MI) and Lake Michigan (St. James Harbor [LM]; Fig. 1) were sampled during three collection periods in the summer of 2014: period 1 (June 10–11), period 2 (July 28–30), and period 3 (Aug. 30–31). Sampling sites for the three inland lakes were located at the region of greatest depth (at 3.6 m for Barney’s Lake, 15.2 m for Lake Geneserath, and 6.1 m for Fox Lake). Lake Michigan bottom sampling depth ranged from 14.5–18.3 m, depending upon small-scale spatial bathymetric differences. While Lake Michigan was not sampled at the point of greatest depth (as were other lakes in this study), we attempted to sample Lake Michigan to a similar depth as inland lakes within this study.

Surface and bottom-water samples were collected using a Kemmerer (Wildco^®^, Yulee, FL, USA) water sampler. During each collection period, samples were retrieved from surface (one meter below the surface) and bottom-water (one meter above the lake sediment) locations for each site. For each sample, water was collected in an acid washed sterile bottle. From this bottle, 90 ml of water was filtered through a 0.45 µm filter (Whatman, GE Healthcare, Little Chalfont, Buckinghamshire, UK) into acidified vials (resulting pH of 3) and stored on ice for DOC analysis. For collection of microbial samples, 120 ml of water was syringe filtered through a combination of two filters (2.2 µm first, followed by 0.22 µm filters). The filters were flash frozen (dry-ice and ethanol bath) in the field, and then stored at −80 °C. Once per sampling period, 120 ml of sterilized Nanopure water was filtered and frozen in the field as a control for microbial samples. The remaining water was stored on ice and then filtered (0.45 µm) in the lab for nutrient analyses.

A calibrated Hydrolab^®^ DS5 (OTT Hydromet, Kempten, Bavaria, Germany) was used to generate a physicochemical profile of each lake prior to sample collection. Measured parameters included dissolved oxygen (percent and mg/L), temperature (°C), and pH (raw data can be found in Table S1).

### Water chemistry analyses

For nutrient analysis, 250 ml of water from each sampling location and depth was filtered in the lab through a 0.45 µm filter (Whatman) and frozen at −20 °C. A Quaatro Bran+Luebbe Auto Analyzer with an XY-2 Sampler (Seal Analytical, Mequon, WI, USA) was used to determine soluble reactive phosphorus (SRP), ammonium (NH_4_), nitrate (NO${}_{3}^{-}$), total nitrogen (TN), and total phosphorus (TP) concentrations in the water. An additional 10 ml of water was filtered (0.45 µm) and acidified for dissolved organic matter (DOM) analysis. Proxies of DOM were characterized by their specific absorption coefficient (SAC340) (Chappaz & Curtis, 2013; Curtis & Adams, 1995) and specific UV absorbance (SUVA254) (Mcknight et al., 2001; Weishaar et al., 2003). Triplicates of each sample were placed into quartz cuvettes (1 cm width) and UV absorbance readings were taken at two different wavelengths: 254 nm and 340 nm. Samples collected for DOC analysis (described above) were quantified using a Shimadzu TOC-V analyzer (Kyoto, Japan). Raw water chemistry data can be found in Table S2.

### Microbial taxonomic analysis

DNA was extracted from frozen filters using the MoBio PowerWater^®^ DNA isolation kit (following the manufacturer’s protocol). DNA was extracted from both .22 and 2.2 µm filters from the same sample simultaneously. All samples were concentrated in a Zymo DNA Clean & Concentrator™ kit before being quantified by a Qubit^®^ 2.0 Fluorometer (Life Technologies, Carlsbad, CA, USA). Control samples yielded DNA that was below detection limits (<0.5 ng/mL). In order to obtain a sufficient amount of DNA for downstream sequencing, PCRs were completed for each sample to amplify the 16S rRNA gene using high-fidelity *Taq* polymerase (New England BioLabs Inc., Ipswich, MA, USA) and 27F and 1492R primers (Weisburg et al., 1991). PCR conditions implemented were as follows: initial denaturation at 95 °C for 5 min, followed by 36–40 cycles (denaturation at 95 °C for 30 s, annealing at 56 °C for 30 s, and extension at 72 °C), and final extension at 72 °C for 10 min. The number of cycles for each sample varied due to differences in amplification (Table S3), which was visualized through gel electrophoresis. Replicate PCRs for each sample were pooled. PCR samples were purified using the QIAquick^^®^^ Gel Extraction Kit (Qiagen, Hilden, North Rhine-Westphalia, Germany). Three sampling points were excluded from microbial community data analysis, which included bottom-water time point “1” for Lake Michigan and both surface and bottom-water community profiles for Fox Lake time point “3”. These samples were excluded, as they either did not contain sufficient concentration or quality of DNA for sequencing or analysis. V4 16S rRNA amplicons were generated using previously described methods and primers 16Sf-V4 (515f) and 16Sr-V4 (806r) (Kozich et al., 2013) and sequenced on an Illumina MiSeq platform using a paired end 2 × 250 bp format (accomplished by Michigan State University’s Research Technology Support Facility).

Sequence data were processed using MOTHUR v.1.35.1 (Schloss et al., 2009). Quality control and clustering steps were implemented following the publicly available MiSeq SOP (found at http://www.mothur.org/) with modifications. Briefly, sequences which were less than 251 bp or greater than 254 bp in length were removed from further analyses, as were sequences which contained >8 homopolymers. Sequences were aligned using the SILVA (v. 119) reference database (Quast et al., 2012). Sequences which were not aligned within the V4 region were also removed. UCHIME (Edgar et al., 2011) was used to check for chimeric DNA, which was subsequently removed. Sequences were classified using the RDP database (training set v9; Cole et al., 2013). Classifications corresponding to chloroplast, eukaryotic, or mitochondrial DNA, as well as sequences that classified as unknown, were removed. The remaining data were clustered into operational taxonomic units (OTUs) using a 0.03 dissimilarity threshold. The Mothur workflow associated with this study can be found within an online repository located on GitHub (https://github.com/horto2dj/CMUBS_microb). Sequences obtained for this study have been deposited in the MG-RAST database (Meyer et al., 2008) under accession numbers mgm4732740.3–mgm4732751.3, mgm4732757.3, mgm4732760.3, mgm4733677.3–mgm4733686.3, mgm4733688.3, mgm4733690.3–mgm4733704.3, and mgm4733784.3–mgm4733785.3. Additional metadata associated with submitted environmental sequences can be found within Table S3.

### Statistical analyses

Statistical analyses (both chemical and biological) were completed using the R statistical software v.3.2.1 (R Core Team, 2015). Protocols and files associated with quality control and statistical tests can be found on GitHub (https://github.com/horto2dj/CMUBS_microb). Differences in lake chemistry among lakes and time points within lakes were analyzed through principal component analysis (PCA).

Prior to alpha and beta diversity analyses, singletons and doubletons were removed and samples were normalized using the *DeSeq2* package (Love, Huber & Anders, 2014) in R, followed by a variance stabilizing transformation (McMurdie & Holmes, 2014).

Using the *PhyloSeq package* (McMurdie & Holmes, 2013), Shannon’s diversity was calculated for microbial communities of each sample. Linear mixed-effect models (with ‘Lake’ as random effect) and ANOVA were used to test significance of habitat (i.e., surface vs bottom-water) on levels of alpha diversity. Linear models and ANOVA were used to test for differences in alpha diversity between lakes. Alpha diversity values were correlated with measured environmental variables using Spearman’s rank correlation to explore relationships between environmental variables and alpha diversity.

Non-metric multidimensional scaling (NMDS) based on Bray–Curtis distance was performed to compare dissimilarity between the samples, also employing the *PhyloSeq* package. A total of 20 iterations were accomplished to reach the lowest stress during NMDS and two dimensions (*k* = 2) were used for visualization. Analysis of Similarity (ANOSIM) was used to test for significant differences in community composition between microbial communities of different lakes. Correlation of environmental variables with microbial communities was determined using *envfit* of the Vegan package (Oksanen et al., 2017). Canonical Correspondence Analysis (CCA) was implemented to explore relationships between environmental variables significantly correlated to beta diversity in NMDS and microbial community beta diversity. Permutation tests were implemented to test significance of axes and environmental variables within CCA in explaining microbial community beta diversity patterns using 999 permutations in all tests. Partial Canonical Correspondence Analysis (pCCA) was implemented to specifically examine potential effects of oxygen gradients on microbial communities in the same way as described above.

Spearman’s Rank correlations were used to identify OTUs significantly correlated to environmental variables (i.e., dissolved oxygen, pH, and temperature). Only OTUs which appeared within a minimum of five samples with at least two sequences were considered for correlation analyses. Variance stabilizing transformation was used to normalize sequence abundances across samples for these OTUs to account for uneven sequencing depth between samples. Correlations with *p* > 0.001 and *r* < 0.65 were excluded as an attempt to reduce spurious correlations. OTUs which could not be identified as belonging to a phylum were removed from analyses.

## Results & Discussion

### Physicochemical variation among and within lakes

**Figure 2:**
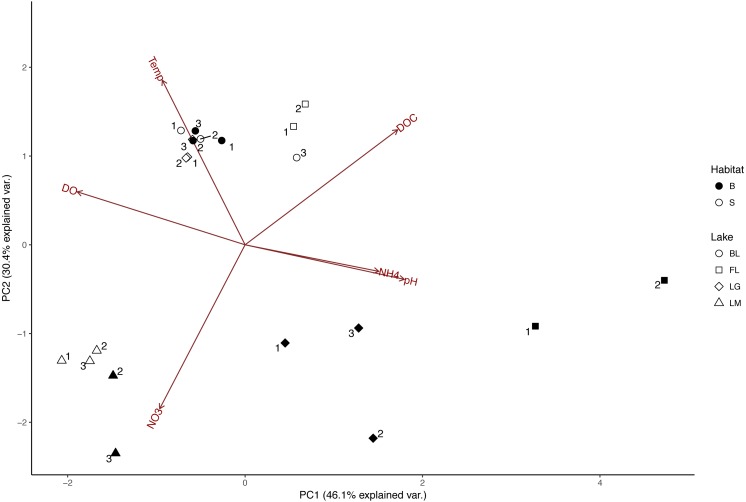
Principal Component Analysis (PCA) demonstrating separation of all sampling points based on measured environmental variables (pH, temperature, nitrate, ammonium, nitrate, dissolved oxygen, and dissolved organic carbon). Circles, Barney’s Lake; squares, Fox Lake; diamonds, Lake Geneserath, triangles, Lake Michigan. Open shapes correspond to surface-water samples, while filled shapes correspond to bottom-water samples. Numbers associated with points correspond to time point of sampling (higher numbers are later in the summer). DO, dissolved oxygen; DOC, dissolved organic carbon; NH4, ammonium; NO3, nitrate; Temp, temperature. SAC340 and NO${}_{3}^{-}$ data were highly correlated (Spearman Rank, *r* = 0.82, *p* < 0.001) so both were represented by “NO_3_” in the PCA.

Fundamental differences in lake physicochemistry were observed between Lake Michigan and inland lakes on Beaver Island (Fig. 2; Table 1). Lake Michigan water chemistry was distinguished based upon DOC, NO${}_{3}^{-}$, and SAC340 concentrations, showing considerable divergence from the remaining three lakes (Fig. 2). Lake surface physicochemistry (temperature, DOC concentrations, and DOM properties) was nearly indistinguishable between Barney’s Lake and Lake Geneserath. Fox Lake surface water chemistry was also similar to that of Barney’s Lake and Lake Geneserath, but was slightly dissimilar due to a lower pH with respect to other lakes.

Of the lakes sampled, Lake Geneserath and Fox Lake experienced oxygen, temperature, and pH stratification over time between surface and bottom-waters (Fig. 2, Table S2). The bottom-water in both lakes experienced lower temperatures, elevated acidity, and lower oxygen levels with respect to the surface-water. DOC concentrations and DOM quality did not vary significantly (two-tailed *t*-test, *p* > 0.05) between surface-water and bottom-water environments for any lake. Barney’s Lake and Lake Michigan did not experience physicochemical stratification at the points sampled. However, Lake Michigan bottom water experienced decreased temperatures, but did not plateau with increasing depth, suggesting that the thermocline rather than the hypolimnion was developed at the sampling location.

**Table 1:** Limnological characteristic ranges for the surface and bottom water of each lake during the duration of this study.

	Habitat	Temp (°C)	pH	DO (%)	DOC (mg/L)
Barney’s Lake	Surface	20.7–21.7	8.57–8.75	103.1–118.7	10.7–11.4
	Bottom	20.4–21.2	8.52–8.75	103.7–113.9	10.6–11.2
Fox Lake	Surface	20.7–21.5	6.13–6.53	90.7–93.8	16.0–17.6
	Bottom	11.1–12.5	5.46–5.79	0–25.4	16.2–18.5
Lake Geneserath	Surface	19.7–21.4	8.05–8.29	97.8–103.4	9.2–9.8
	Bottom	8.8–9.1	6.49–6.78	0–64.0	9.0–9.2
Lake Michigan	Surface	16.1–18.2	8.12–8.28	102.1–124.5	2.4–2.8
	Bottom	8.1–15.9	7.84–8.08	103.4–120	2.1–2.6

**Notes.**

Temp temperature DO dissolved oxygen DOC dissolved organic carbon

### Microbial community taxonomy and alpha diversity among lakes

A total of 3,415,100 sequences were obtained across all samples prior to filtering and quality control steps. After quality filtering steps, 2,058,143 sequences remained and from these sequences 51,831 OTUs were identified. Sequencing depth ranged from 54,802 to 136,518 total sequences among samples. After singletons and doubletons were removed, a total of 20,372 OTUs remained for diversity analyses. There were no significant differences in alpha diversity among lakes according to linear models and ANOVA. However, linear mixed-effect models and ANOVA found that habitat type (i.e., surface vs bottom-water) significantly influenced Shannon diversity levels (*p* < 0.01), with higher levels of diversity occurring in bottom-water habitat versus the surface-water (Table 2). Previous literature that suggests anoxic hypolimnion communities are more diverse (alpha diversity) than their respective epilimnion (Humayoun, Bano & Hollibaugh, 2003; Shade et al., 2012b; Meyerhof et al., 2016), which is consistent with our findings in lakes which developed anoxic hypolimnia (Fox Lake and Lake Geneserath). Two of the lakes within this study, Barney’s Lake and Lake Michigan, did not develop anoxic hypolimnia, yet these systems experienced higher alpha diversity in their bottom-water environments with respect to surface waters. These differences in alpha diversity (namely evenness) between surface and bottom-water environments may be driven by other variables, such as temperature (in Lake Michigan), or other variables not measured in this study, such as light penetration. A separate study exploring microbial communities along a Lake Michigan transect south of our sampling location did not find differences in alpha diversity between epilimnion and hypolimnion environments (Fujimoto et al., 2016). However, as we did not sample the hypolimnion of Lake Michigan in our study, our results are not directly comparable to the findings of Fujimoto et al. (2016). Despite this, we found differences between Lake Michigan epilimnion and thermocline environments, which suggests potentially higher diversity within the thermocline with respect to the surface water environment.

**Table 2:** Shannon diversity values for microbial communities from each collection point.

Lake	Habitat	Time	Shannon
BL	B	1	4.21
		2	4.83
		3	4.32
	S	1	3.59
		2	4.07
		3	4.27
FL	B	1	4.19
		2	4.91
	S	1	3.22
		2	4.72
LG	B	1	4.01
		2	4.33
		3	4.67
	S	1	3.73
		2	4.7
		3	4.29
LM	B	2	4.49
		3	4.91
	S	1	3.48
		2	3.74
		3	3.18

**Figure 3:**
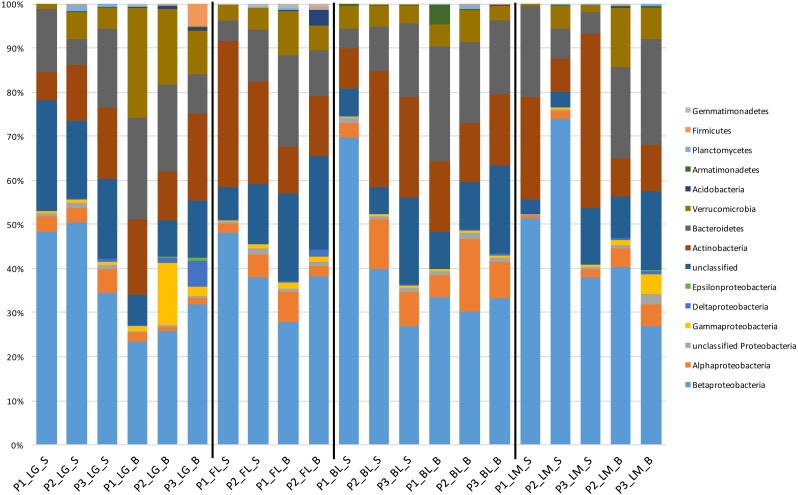
Taxonomic composition and relative abundance (>1% relative abundance) of community members broken down by phylum at each sampling location. Labeling scheme is represented by lake (e.g., LG, FL, BL, and LM), followed by habitat (e.g., surface-water [S] and bottom-water [B]), and finally sampling time point (e.g., P1, sampling time 1, P2, sampling time 2, etc.).

Taxonomically, members of *Proteobacteria*, primarily *Betaproteobacteria*, were generally the most dominant taxa (based on relative abundance) found within the sequenced microbial community in all the explored lakes (Fig. 3). Other dominant phyla (>1% community composition) within the lake systems included *Acidobacteria, Actinobacteria*, *Armatimonadetes*, *Bacteroidetes*, *Firmicutes*, G*ammatimonadetes*, *Planctomycetes*, and *Verrucomicrobia*. These phyla have frequently been shown to dominate freshwater communities (Attermeyer et al., 2015; Boucher, Jardillier & Debroas, 2006; Taipale, Jones & Tiirola, 2009; Zwart et al., 2002). The most abundant OTU within the inland lakes, and second most abundant in Lake Michigan, was related to *Polynucleobacter* within *Betaproteobacteria*. This microbial genus has been commonly found in freshwater systems, with levels up to 60% community composition found in one freshwater pond (Hahn, 2003; Hahn, Pockl & Wu, 2005; Hahn et al., 2010; Jezbera et al., 2011) and represented the third most dominant OTU of another stratified lake (Garcia et al., 2013).

**Figure 4:**
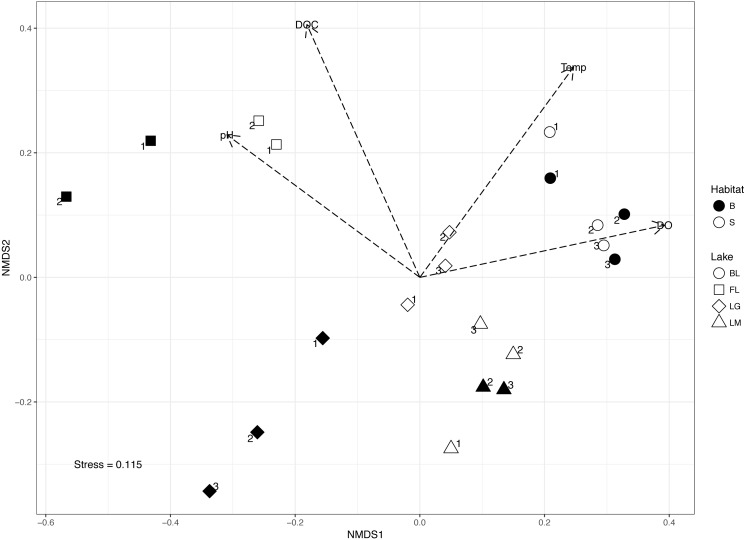
Non-metric Multidimensional Scaling (NMDS) of microbial communities. NMDS of microbial communities within each sampled lake. Circles, Barney’s Lake; squares, Fox Lake; diamonds, Lake Geneserath, triangles, Lake Michigan. Open shapes correspond to surface-water samples, while filled shapes correspond to bottom-water samples. Numbers associated with points correspond to time point of sampling. Vectors correspond to environmental variables significantly correlated (*p* < 0.001, *R* > 0.5) to separation of microbial communities within NMDS. DO, dissolved oxygen; DOC, dissolved organic carbon; Temp, temperature.

### Environmental relationships with microbial beta diversity

Beta diversity ordinations incorporating all sites showed microbial communities separated based on the sampling location (or lake) (Fig. 4; ANOSIM *R* = 0.789, *p* = 0.001). Significant relationships (*p* < 0.001) were found between environmental conditions and microbial beta diversity, including correlations between community structure and dissolved oxygen (*r* = 0.645), dissolved organic carbon (*r* = 0.790), pH (*r* = 0.593), and temperature (*r* = 0.699). Environmental variables found to significantly correlate to beta diversity in NMDS (i.e., DO, DOC, pH, and temperature) were tested as constraining variables on beta diversity. CCA was found to be significant (*F* = 1.4245, *p* < 0.001) in explaining microbial beta diversity among all samples (Fig. S1). Constraining variables explained 26.26% of variation in CCA. CCA1 and CCA2 were both significant (*p* < 0.001), explaining 31.28% and 28.56% of constrained variation, respectively. DO and pH were significant constraints on microbial community beta diversity (*p* < 0.001), as was temperature to a lesser degree (*p* < 0.05). DOC, however, was not found to be significantly related to microbial community beta diversity. Similarly, Jones, Newton & McMahon (2009) found that DOC concentration does not predict microbial community structural differences, but rather quality of organic carbon (as measured by water color: chlorophyll-a) is significantly related to microbial community structure in freshwater lakes. The influence of DO on microbial community structure is of particular interest due to oxygen’s influence on regulation of redox cycles within aquatic systems. As such, partial CCA (pCCA) examining the strength of dissolved oxygen as an environmental constraint on microbial community structure was accomplished while controlling for temperature and pH within sampled lakes. Partial CCA found that oxygen alone was significantly related to microbial community composition (*p* < 0.001, Fig. S2) irrespective of the influence of pH and temperature. The lakes sampled within our study were all located on (or near) Beaver Island within 17 km of each other, yet they were physicochemically diverse, suggesting that environmental constraints on microbial communities are stronger than geographic distance. These results are consistent with established theory that microbial community structure and taxa can be highly constrained by environmental factors within lakes, while geographic proximity of lakes may explain to a lesser degree microbial community structure (Yannarell & Triplett, 2005; Van der Gucht et al., 2007).

Surface and bottom-water microbial communities within lakes that experienced oxygen and pH stratification (Lake Geneserath and Fox Lake) separated over time (Fig. 4). These results are consistent with previous research that has found divergence of microbial community beta diversity between epilimnia and hypolimnia after lake mixing events (Shade, Chiu & McMahon, 2010a), particularly in relation to differences in oxygen as a strong constraint ( Shade, Jones & McMahon, 2008; Shade et al., 2011). Interestingly, while surface-water microbial communities remained relatively stable within these stratifying lakes, bottom-water communities showed marked divergence over time. Previous studies have illustrated that hypolimnetic communities are not resistant to disturbances, particularly disturbances related to oxygen or key nutrient shifts (Allison & Martiny, 2008; Shade et al., 2011; Shade et al., 2012b). Our results corroborate that lake stratification may be an important factor in shaping these communities across freshwater lakes which experience water column mixing events. Contrastingly, within lakes which did not experience stratification, community composition was indistinguishable between surface-water and bottom-water communities within each lake respectively. Previous research has found that microbial communities within oxygenated hypolimnia of Lake Michigan and other deep lakes are often structurally distinct from the respective epilimnia (Fujimoto et al., 2016; Okazaki et al., 2017). It is likely that we did not find distinctness between surface water and bottom water communities of Lake Michigan, as the hypolimnion of Lake Michigan was not sampled within this study. The Lake Michigan sampling point was also shallower in depth than locations explored by Okazaki et al. (2017) and a separate location than studied by Fujimoto et al. (2016).

**Figure 5:**
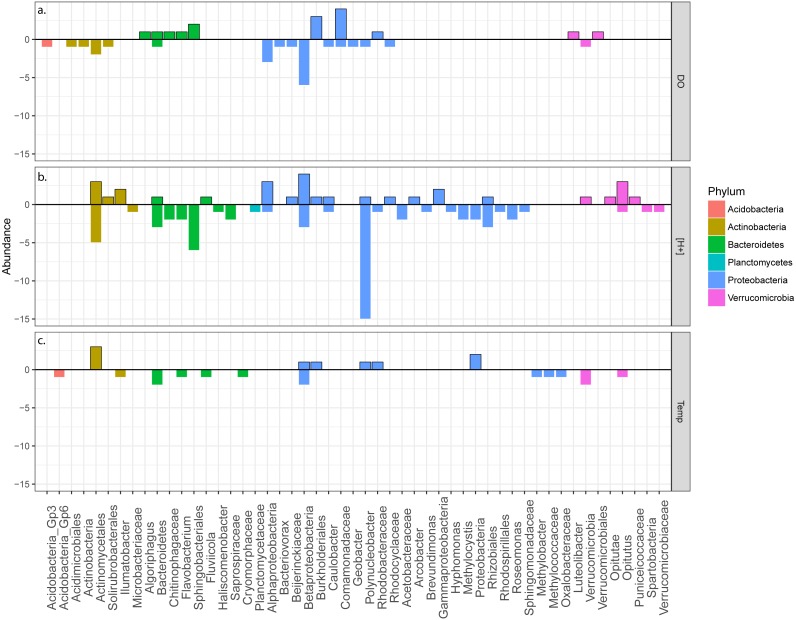
Bar plot of taxonomic groups. Bar plot illustrating correlations of taxonomic groups (identified to their lowest phylogenetic classification) to environmental variables (A) dissolved oxygen (DO), (B) pH ([H +]), and (C) temperature (Temp). Abundance values indicate number of OTUs identified to a taxonomic group either positively or negatively correlated to an environmental variable. Values above the “0” line indicate positively correlated OTU abundances, while values below this same line represent negatively correlated OTU abundances. Bar colors correspond to the phylum each lower taxonomic group belongs to.

### Taxonomic relationships to environmental variables

Nine hundred twenty-eight microbial OTUs were found within a minimum of five samples and these shared OTUs were analyzed for correlations with measured environmental variables. In general, specific taxonomic groups (at the level of genus or higher) appeared to be either positively or negatively correlated to levels of dissolved oxygen, as there was little contrast in correlation direction from the same taxonomic group (Fig. 5A). Specifically, members of the phylum *Bacteroidetes* were primarily positively correlated with dissolved oxygen (*n* = 6), where only one *Bacteroidetes* OTU was negatively related to dissolved oxygen. Within *Bacteroidetes*, a representative OTU from the genus *Algoriphagus*, which has been described as a strict aerobe (Bowman, Nichols & Gibson, 2003; Liu et al., 2009), was found to positively correlate with dissolved oxygen concentrations. Members of *Flavobacteria* have been known to be primarily aerobic (Bernardet et al., 1996), and were also found to positively correlate to dissolved oxygen concentration. Other OTUs, related to *Sphingobacteriales* (including *Chitinophagaceae*), contain representative aerobic microbial taxa (Rosenberg, 2014) and are common in freshwater bodies within the Great Lakes basin (Mou et al., 2013), so it is not surprising to find these taxa within aerobic freshwater environments within the temperate freshwater lakes of the Great Lakes region. *Comamonadaceae* were generally positively related with dissolved oxygen levels, however, one representative was negatively correlated. Research has demonstrated that some members of this primarily aerobic family indeed can grow under anaerobic conditions (Ramana & Sasikala, 2009). Many OTUs related to the order *Burkholderiales* also increased with oxygen availability. It is likely that these taxa are unable to adapt to developing anoxia within the hypolimnion of chemically stratifying lakes, and may play more dominant roles within the epilimnion after stratification has occurred post-mixing.

To the contrary, several individual OTUs negatively correlated with dissolved oxygen. Members of the phyla *Acidobacteria*, *Actinobacteria*, *Alphaproteobacteria*, and *Deltaproteobacteria* only had representative OTUs found to be inversely correlated to dissolved oxygen concentrations. Representative OTUs from *Alphaproteobacteria* included taxa related to *Caulobacter* and *Rhodocyclaceae*, both of which are bacteria that could thrive under anaerobic conditions (Song et al., 2013; Oren, 2014). From *Deltaproteobacteria*, *Geobacter*, a well-renowned anaerobe (Lovley & Phillips, 1988; Lovley et al., 1999), was found to be negatively correlated to oxygen, as was *Bacteriovorax*, of which much less is known regarding its metabolism in freshwater systems. These results point towards taxa that may prosper in developing anaerobic hypolimnetic environments after a lake mixing event has disturbed the water column.

Frequently, OTUs were idiosyncratic in their relationships to higher or lower pH levels within the same Phylum and ranging down to Genus (Fig. 5B). This suggests that preferences for ideal environmental pH are often at the level of OTU, and generalizations cannot be drawn for many taxonomic groups. Despite this, there were groups of bacteria that correlated predominantly with decreasing [H^+^], with few or no representative OTUs correlating with increasing [H^+^]. For example, *Polynucleobacter* OTUs almost resoundingly correlated to decreasing [H^+^], despite previous research suggesting that members within this genus comprise a higher proportion of microbial communities within environments characterized by circumneutral to acidic pH (Jezbera et al., 2012). It is possible that these taxa may have been constrained by other factors (such as DOM or lack of O_2_), which limited them from thriving within lower pH environments often corresponding with lower O_2_ levels. From the phylum *Bacteroidetes*, OTUs related to *Chitinophagaceae*, *Flavobacterium*, and *Sphingobacteriales* negatively correlated to [H^+^], as did *Proteobacteria* members such as *Acetobacteraceae*, *Hyphomonas*, *Methylobacter*, and *Roseomonas*. As pH generally decreases with increasing depth within a water column, it could be superficially suggested that these taxa may be more abundant in shallower depths of the water column. As an example, *Bacteroidetes* which negatively correlated to [H^+^] contained OTUs which positively correlated to dissolved oxygen, suggesting that these OTUs are likely present within epilimnia of stratified lakes. Interestingly, the family *Acetobacteraceae,* which contains members of the acetic acid bacteria (including *Roseomonas*), are often adapted to lower pH levels due to their ability to produce acetic acid during metabolism (Raspor & Goranovič, 2008). However, members within this group are obligate aerobes (Raspor & Goranovič, 2008), and thus may have been unable to tolerate lower O_2_ conditions as may have been the case for OTUs related to *Polynucleobacter*.

Temperature did not appear to have a large influence on individual microbial taxa within these lakes relative to the potential influences of pH and dissolved oxygen (Fig. 5C). However, individual OTUs spread across several phyla periodically correlated with temperature either positively or negatively. Most notably, *Actinomycetales*, which possess thermophilic taxa (Korn-Wendisch et al., 1995), contained three OTUs which positively correlated to temperature, suggesting that these taxa may be most prevalent in shallow lakes or epilimnia.

## Conclusion

This study has found that microbial communities within actively physicochemically stratifying lakes, particularly stratification of dissolved oxygen, pH, and temperature, diverge to a larger degree over time relative to communities within lakes (or points within lakes) that do not chemically stratify. Additionally, despite their relatively close geographic proximity, each lake harbored a distinct microbial community, suggesting that lake physicochemistry is a stronger constraint on microbial communities than geographic region. Correlations of individual microbial OTUs to physical and chemical variables, such as dissolved oxygen, pH, and temperature, could be related to metabolic capabilities of microbial taxonomic groups or individual OTUs. This suggests that lake stratification and environmental conditions unique to each lake may influence the prevalence of some microbial taxa more strongly than others, thereby potentially influencing ecosystem processes carried out by these taxa. This research highlights the importance of sampling lakes in the same geographic area but distinct in physical and chemical attributes, as well as the potential impact of lake mixing and stratification as a disturbance to microbial communities within temperate freshwater lake systems, which could ultimately influence microbial community functional diversity and biogeochemical processes.

##  Supplemental Information

10.7717/peerj.3937/supp-1 Figure S1 Canonical Correspondence Analysis (CCA) of microbial communities across lakes Circles , Barney’s Lake, squares , Fox Lake, diamonds = Lake Geneserath, triangles , Lake Michigan. Open shapes correspond to surface-water samples, while filled shapes correspond to bottom-water samples. Numbers associated with points correspond to time point of sampling. Vectors correspond to environmental variables used to constrain variability in CCA. DO, dissolved oxygen; DOC, dissolved organic carbon; Temp, temperature. Percentages associated with axes correspond to percent constrained variability explained.

10.7717/peerj.3937/supp-2 Figure S2 Partial Canonical Correspondence Analysis (pCCA) of microbial communities Partial Canonical Correspondence Analysis (pCCA) of microbial communities across lakes with oxygen as the constraining variable while controlling for variability caused by temperature and pH. Circles , Barney’s Lake, squares , Fox Lake, diamonds = Lake Geneserath, triangles , Lake Michigan. Open shapes correspond to surface-water samples, while filled shapes correspond to bottom-water samples. Numbers associated with points correspond to time point of sampling. Vectors correspond to environmental variables used to constrain variability in CCA. DO, dissolved oxygen. Percentages associated with axes correspond to percent total variability explained.

10.7717/peerj.3937/supp-3 Table S1 Hydrolab depth profiles for each lake and sampling period

10.7717/peerj.3937/supp-4 Table S2 Summary of Hydrolab and nutrient data collected at each sampling location DO, dissolved oxygen, SRP, soluble reactive phosphorus, TN, total nitrogen, and TP, total phosphorus, DOC, Dissolved Organic Carbon.

10.7717/peerj.3937/supp-5 Table S3 Metadata associated with environmental samples analyzed within this study

## Additional Information and Declarations

### DNA Deposition

The following information was supplied regarding the deposition of DNA sequences: Sequences obtained for this study have been deposited in the MG-RAST database (Meyer et al., 2008) under accession numbers mgm4732740.3–mgm4732751.3, mgm4732757.3, mgm4732760.3, mgm4733677.3–mgm4733686.3, mgm4733688.3, mgm4733690.3–mgm4733704.3, and mgm4733784.3–mgm4733785.3.

### Data Availability

The following information was supplied regarding data availability: Github: https://github.com/horto2dj/CMUBS_microb.

## References

[ref-1] Allison SD, Martiny JB (2008). Resistance, resilience, and redundancy in microbial communities. Proceedings of the National Academy of Sciences of the United States of America.

[ref-2] Amaral V, Graeber D, Calliari D (2016). Strong linkages between DOM optical properties and main clades of aquatic bacteria. Limnology and Oceanography.

[ref-3] Andrei A, Robeson MS, Baricz A, Coman C, Muntean V, Ionescu A, Etiope G, Alexe M, Sicora CI, Podar M (2015). Contrasting taxonomic stratification of microbial communities in two hypersaline meromictic lakes. ISME Journal.

[ref-4] Attermeyer K, Tittel J, Allgaier M, Frindte K, Wurzbacher C, Hilt S, Kamjunke N (2015). Effects of light and autochthonous carbon additions on microbial turnover of allochthonous organic carbon and community composition. Microbial Ecology.

[ref-5] Bernardet J, Segers P, Vancanneyt M, Berthe F, Kersters K (1996). Cutting a gordian knot: emended classification and description of the genus flavobacterium, emended description of the family flavobacteriaceae, and proposal of flavobacterium hydatis nom. nov. (Basonym, Cytophaga aquatilis Strohl and Tait 1978). International Journal of Systematic Bacteriology.

[ref-6] Bier RL, Bernhardt ES, Boot CM, Graham EB, Hall EK, Lennon JT, Nemergut DR, Osborne BB, Ruiz-Gonzalez C, Schimel JP, Waldrop MP, Wallenstein MD (2015). Linking microbial community structure and microbial processes: an empirical and conceptual overview. FEMS Microbiology Ecology.

[ref-7] Boucher D, Jardillier L, Debroas D (2006). Succession of bacterial community composition over two consecutive years in two aquatic systems: a natural lake and a lake-reservoir. FEMS Microbiology Ecology.

[ref-8] Bowman JP, Nichols CM, Gibson  JA (2003). Algoriphagus ratkowskyi gen. nov. sp. nov. Brumimicrobium glaciale gen. nov. sp. nov. Cryomorpha ignava gen. nov. sp. nov. and Crocinitomix catalasitica gen. nov. sp. nov. novel flavobacteria isolated from various polar habitats. International Journal of Systematic and Evolutionary Microbiology.

[ref-9] Burkert U, Warnecke F, Babenzien D, Zwirnmann E, Pernthaler  J (2003). Members of a readily enriched -proteobacterial clade are common in surface waters of a humic lake. Applied and Environmental Microbiology.

[ref-10] Chappaz A, Curtis PJ (2013). Integrating empirically dissolved organic matter quality for WHAM VI using the DOM optical properties: a case study of Cu–Al–DOM interactions. Environmental Science and Technology.

[ref-11] Clement TA, Murry BA, Uzarski DG (2015). Fish community size structure of small lakes: the role of lake size, biodiversity and disturbance. Journal of Freshwater Ecology.

[ref-12] Cole JR, Wang Q, Fish JA, Chai B, Mcgarrell DM, Sun Y, Brown CT, Porras-Alfaro A, Kuske CR, Tiedje  JM (2013). Ribosomal Database Project: data and tools for high throughput rRNA analysis. Nucleic Acids Research.

[ref-13] Cotner JB, Biddanda BA (2002). Small players, large role: microbial influence on biogeochemical processes in pelagic aquatic ecosystems. Ecosystems.

[ref-14] Crump BC, Kling GW, Bahr M, Hobbie JE (2003). Bacterioplankton community shifts in an arctic lake correlate with seasonal changes in organic matter source. Applied and Environmental Microbiology.

[ref-15] Curtis PJ,  Adams  HE (1995). Dissolved organic matter quantity and quality from freshwater and saltwater lakes in east-central Alberta. Biogeochemistry.

[ref-16] Edgar RC, Haas BJ, Clemente JC, Quince C, Knight R (2011). UCHIME improves sensitivity and speed of chimera detection. Bioinformatics.

[ref-17] Eiler A, Langenheder S, Bertilsson S, Tranvik  LJ (2003). Heterotrophic bacterial growth efficiency and community structure at different natural organic carbon concentrations. Applied and Environmental Microbiology.

[ref-18] Essington TE, Carpenter SR (2000). Mini-review: nutrient cycling in lakes and streams: Insights from a comparative analysis. Ecosystems.

[ref-19] Fenchel T, Finlay  B (2008). Oxygen and the spatial structure of microbial communities. Biological Reviews.

[ref-20] Fujimoto M, Cavaletto J, Liebig JR, McCarthy A, Vanderploeg HA, Denef VJ (2016). Spatiotemporal distribution of bacterioplankton functional groups along a freshwater estuary to pelagic gradient in Lake Michigan. Journal of Great Lakes Research.

[ref-21] Garcia SL, Salka I, Grossart H, Warnecke F (2013). Depth-discrete profiles of bacterial communities reveal pronounced spatio-temporal dynamics related to lake stratification. Environmental Microbiology Reports.

[ref-22] Grossart H, Jezbera J, Horňák K, Hutalle KM, Buck U, Šimek  K (2008). Top-down and bottom-up induced shifts in bacterial abundance, production and community composition in an experimentally divided humic lake. Environmental Microbiology.

[ref-23] Hahn MW (2003). Isolation of strains belonging to the cosmopolitan polynucleobacter necessarius cluster from freshwater habitats located in three climatic zones. Applied and Environmental Microbiology.

[ref-24] Hahn MW, Lang E, Brandt U, Lunsdorf H, Wu QL, Stackebrandt  E (2010). Polynucleobacter cosmopolitanus sp. nov. free-living planktonic bacteria inhabiting freshwater lakes and rivers. International Journal of Systematic and Evolutionary Microbiology.

[ref-25] Hahn MW, Pockl M, Wu  QL (2005). Low intraspecific diversity in a polynucleobacter subcluster population numerically dominating bacterioplankton of a freshwater pond. Applied and Environmental Microbiology.

[ref-26] Humayoun SB, Bano N,  Hollibaugh  JT (2003). Depth distribution of microbial diversity in Mono Lake, a meromictic soda lake in California. Applied and Environmental Microbiology.

[ref-27] Jezbera J, Jezberová J, Brandt U, Hahn  MW (2011). Ubiquity of Polynucleobacter necessarius subspecies asymbioticus results from ecological diversification. Environmental Microbiology.

[ref-28] Jezbera J, Jezberová J, Koll U, Horňák K, Šimek K, Hahn  MW (2012). Contrasting trends in distribution of four major planktonic betaproteobacterial groups along a pH gradient of epilimnia of 72 freshwater habitats. FEMS Microbiology Ecology.

[ref-29] Jones SE, Newton RJ, McMahon KD (2009). Evidence for structuring of bacterial community composition by organic carbon source in temperate lakes. Environmental Microbiology.

[ref-30] Kim C, Nishimura Y, Nagata T (2006). Role of dissolved organic matter in hypolimnetic mineralization of carbon and nitrogen in a large, monomictic lake. Limnology and Oceanography.

[ref-31] Korn-Wendisch F, Rainey F, Kroppenstedt RM, Kempf A, Majazza A, Kutzner HJ,  Stackebrandt  E (1995). Thermocrispum gen. nov. a new genus of the order actinomycetales, and description of Thermocrispum municipale sp. nov. and Thermocrispum agreste sp. nov. International Journal of Systematic and Evolutionary Microbiology.

[ref-32] Kozich JJ, Westcott SL, Baxter NT, Highlander SK, Schloss  PD (2013). Development of a dual-index sequencing strategy and curation pipeline for analyzing amplicon sequence data on the MiSeq illumina sequencing platform. Applied and Environmental Microbiology.

[ref-33] Li W, Wu F, Liu C, Fu P, Wang J, Mei Y, Wang LY, Guo  JY (2008). Temporal and spatial distributions of dissolved organic carbon and nitrogen in two small lakes on the Southwestern China Plateau. Limnology.

[ref-34] Liu Y, Li H, Jiang JT, Liu YH, Song XF, Xu CJ, Liu  ZP (2009). Algoriphagus aquatilis sp. nov. isolated from a freshwater lake. International Journal of Systematic and Evolutionary Microbiology.

[ref-35] Love MI, Huber W, Anders  S (2014). Moderated estimation of fold change and dispersion for RNA-Seq data with DESeq2. Genome Biology.

[ref-36] Lovley DR, Phillips E (1988). Novel mode of microbial energy metabolism: organic carbon oxidation coupled to dissimilatory reduction of iron or manganese. Applied and Environmental Microbiology.

[ref-37] Lovley DR, Fraga JL, Coates JD, Blunt-Harris  EL (1999). Humics as an electron donor for anaerobic respiration. Environmental Microbiology.

[ref-38] Lucas J, Koester I, Wichels A, Niggemann J, Dittmar T, Callies U, Wiltshire KH, Gerdts  G (2016). Short-term dynamics of north sea bacterioplankton-dissolved organic matter coherence on molecular level. Frontiers in Microbiology.

[ref-39] Mcknight DM, Boyer EW, Westerhoff PK, Doran PT, Kulbe T, Andersen  DT (2001). Spectrofluorometric characterization of dissolved organic matter for indication of precursor organic material and aromaticity. Limnology and Oceanography.

[ref-40] McMurdie PJ, Holmes S (2013). Phyloseq: an R package for reproducible interactive analysis and graphics of microbiome census data. PLOS ONE.

[ref-41] McMurdie PJ, Holmes S (2014). Waste not, want not: why rarefying microbiome data is inadmissible. PLOS Computational Biology.

[ref-42] Meuser JE, Baxter BK, Spear JR, Peters JW, Posewitz MC, Boyd  ES (2013). Contrasting patterns of community assembly in the stratified water column of Great Salt Lake, Utah. Microbial Ecology.

[ref-43] Meyer F, Paarmann D, D’souza M, Olson R, Glass E, Kubal M, Paczian T, Rodriguez A, Stevens R, Wilke A, Wilkening J,  Edwards  R (2008). The metagenomics RAST server—a public resource for the automatic phylogenetic and functional analysis of metagenomes. BMC Bioinformatics.

[ref-44] Meyerhof MS, Wilson JM, Dawson MN, Michael Beman  J (2016). Microbial community diversity, structure and assembly across oxygen gradients in meromictic marine lakes, Palau. Environmental Microbiology.

[ref-45] Mostofa KM, Yoshioka T, Konohira E, Tanoue E, Hayakawa K, Takahashi  M (2005). Three-dimensional fluorescence as a tool for investigating the dynamics of dissolved organic matter in the Lake Biwa watershed. Limnology.

[ref-46] Mou X, Jacob J, Lu X, Robbins S, Sun S, Ortiz  JD (2013). Diversity and distribution of free-living and particle-associated bacterioplankton in Sandusky Bay and adjacent waters of Lake Erie Western Basin. Journal of Great Lakes Research.

[ref-47] Okazaki Y, Fujinaga S, Tanaka A, Kohzu A, Oyagi H, Nakano  SI (2017). Ubiquity and quantitative significance of bacterioplankton lineages inhabiting the oxygenated hypolimnion of deep freshwater lakes. ISME Journal.

[ref-48] Oksanen J, Blanchet FG, Friendly M, Kindt R, Legendre P, McGlinn D, Minchin R, O’Hara RB, Simpson GL, Solymos P, Stevens MHH, Szoecs ED, Wagner H (2017). http://CRAN.R-project.org/package=vegan.

[ref-49] Oren A (2014). The family rhodocyclaceae. The prokaryotes.

[ref-50] Paerl HW, Pinckney JL (1996). A mini-review of microbial consortia: their roles in aquatic production and biogeochemical cycling. Microbial Ecology.

[ref-51] Pilcher DJ, McKinley GA, Bootsma HA, Bennington  V (2015). Physical and biogeochemical mechanisms of internal carbon cycling in Lake Michigan. Journal of Geophysical Research: Oceans.

[ref-52] Quast C, Pruesse E, Yilmaz P, Gerken J, Schweer T, Yarza P, Peplies J, Glöckner FO (2012). The SILVA ribosomal RNA gene database project: improved data processing and web-based tools. Nucleic Acids Research.

[ref-53] R Core Team (2015). https://www.R-project.org/.

[ref-54] Ramana CV, Sasikala C (2009). Albidoferax, a new genus of Comamonadaceae and reclassification of Rhodoferax ferrireducens (Finneran, others, 2003) as Albidoferax ferrireducens comb. nov. Journal of General and Applied Microbiology.

[ref-55] Raspor P, Goranovič D (2008). Biotechnological applications of acetic acid bacteria. Critical Reviews in Biotechnology.

[ref-56] Rosenberg E (2014). The family Chitinophagaceae. The prokaryotes.

[ref-57] Schloss PD, Westcott SL, Ryabin T, Hall JR, Hartmann M, Hollister EB, Lesniewski RA, Oakley BB, Parks DH, Robinson CJ, Sahl JW, Stres B, Thallinger GG, Van Horn DJ, Weber  CF (2009). Introducing mothur: open-source, platform-independent, community-supported software for describing and comparing microbial communities. Applied and Environmental Microbiology.

[ref-58] Shade A, Chiu C, McMahon  KD (2010a). Seasonal and episodic lake mixing stimulate differential planktonic bacterial dynamics. Microbial Ecology.

[ref-59] Shade A, Chiu C, McMahon  KD (2010b). Differential bacterial dynamics promote emergent community robustness to lake mixing: an epilimnion to hypolimnion transplant experiment. Environmental Microbiology.

[ref-60] Shade A, Jones SE, McMahon  KD (2008). The influence of habitat heterogeneity on freshwater bacterial community composition and dynamics. Environmental Microbiology.

[ref-61] Shade A, Peter H, Allison SD, Baho DL, Berga M, Bürgmann H, Huber DH, Langengeder S, Lennon JT, Martiny JBH, Matulich KL, Schmidt TM, Handelsman J (2012a). Fundamentals of microbial community resistance and resilience. Frontiers in Microbiology.

[ref-62] Shade A, Read JS, Welkie DG, Kratz TK, Wu CH, McMahon KD (2011). Resistance, resilience and recovery: aquatic bacterial dynamics after water column disturbance. Environmental Microbiology.

[ref-63] Shade A, Read JS, Youngblut ND, Fierer N, Knight R, Kratz TK, Lottig NR, Roden EE, Stanley EH, Stombaugh J, Whitaker RJ, Wu CH, McMahon KD (2012b). Lake microbial communities are resilient after a whole-ecosystem disturbance. ISME Journal.

[ref-64] Song N, Cai HY, Yan ZS, Jiang  HL (2013). Cellulose degradation by one mesophilic strain Caulobacter sp. FMC1 under both aerobic and anaerobic conditions. Bioresource Technology.

[ref-65] Taipale S, Jones R, Tiirola  M (2009). Vertical diversity of bacteria in an oxygen-stratified humic lake, evaluated using DNA and phospholipid analyses. Aquatic Microbial Ecology.

[ref-66] Tammert H, Kisand V, Nõges  T (2005). Bacterioplankton abundance and activity in a small hypertrophic stratified lake. Hydrobiologia.

[ref-67] Tõnno I, Ott K, Nõges  T (2005). Nitrogen dynamics in the steeply stratified, temperate Lake Verevi, Estonia. Hydrobiologia.

[ref-68] Van der Gucht K, Cottenie K, Muylaert K, Vloemans N, Cousin S, Declerck S, Jeppesen E, Conde-Porcuna J, Schwenk K, Zwart G, Degans H, Vyverman W (2007). The power of species sorting: local factors drive bacterial community composition over a wide range of spatial scales. Proceedings of the National Academy of Sciences of the United States of America.

[ref-69] Weisburg WG, Barns SM, Pelletier DA, Lane  DJ (1991). 16S ribosomal DNA amplification for phylogenetic study. Journal of Bacteriology.

[ref-70] Weishaar JL, Aiken GR, Bergamaschi BA, Fram MS, Fujii R, Mopper  K (2003). Evaluation of specific ultraviolet absorbance as an indicator of the chemical composition and reactivity of dissolved organic carbon. Environmental Science and Technology.

[ref-71] Wetzel RG (2001). Limnology: lake and river ecosystems.

[ref-72] Yannarell AC, Triplett EW (2005). Geographic and environmental sources of variation in lake bacterial community composition. Applied and Environmental Microbiology.

[ref-73] Zadereev E, Tolomeev A, Drobotov  A (2014). Spatial and seasonal dynamics of dissolved and suspended nutrients in the water column of Meromictic Lake Shira. Acta Geologica Sinica.

[ref-74] Zwart G, Crump B, Agterveld MK, Hagen F, Han  S (2002). Typical freshwater bacteria: an analysis of available 16S rRNA gene sequences from plankton of lakes and rivers. Aquatic Microbial Ecology.

