# Supplementary figures and images for: Microbial community diversity patterns are related to physical and chemical differences among temperate lakes near Beaver Island, MI
Source: PeerJ. 2017 Oct 16;5:e3937. doi: 10.7717/peerj.3937 (PMC5647861; doi:10.7717/peerj.3937)

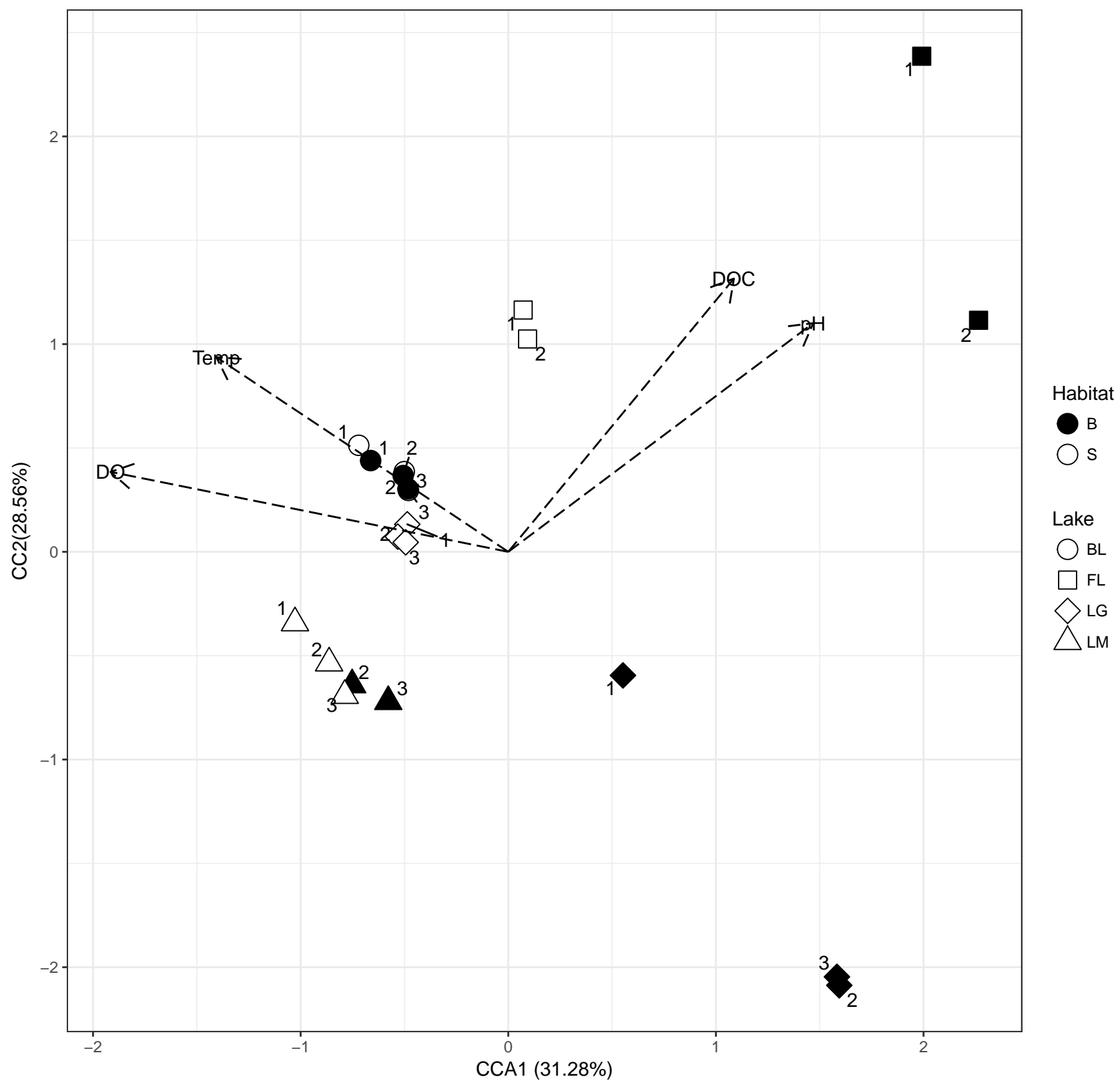

Supplement: Figure S1 — Circles , Barney’s Lake, squares , Fox Lake, diamonds = Lake Geneserath, triangles , Lake Michigan. Open shapes correspond to surface-water samples, while filled shapes correspond to bottom-water samples. Numbers associated with points correspond to time point of sampling. Vectors correspond to environmental variables used to constrain variability in CCA. DO, dissolved oxygen; DOC, dissolved organic carbon; Temp, temperature. Percentages associated with axes correspond to percent constrained variability explained. [file peerj-05-3937-s001.pdf]

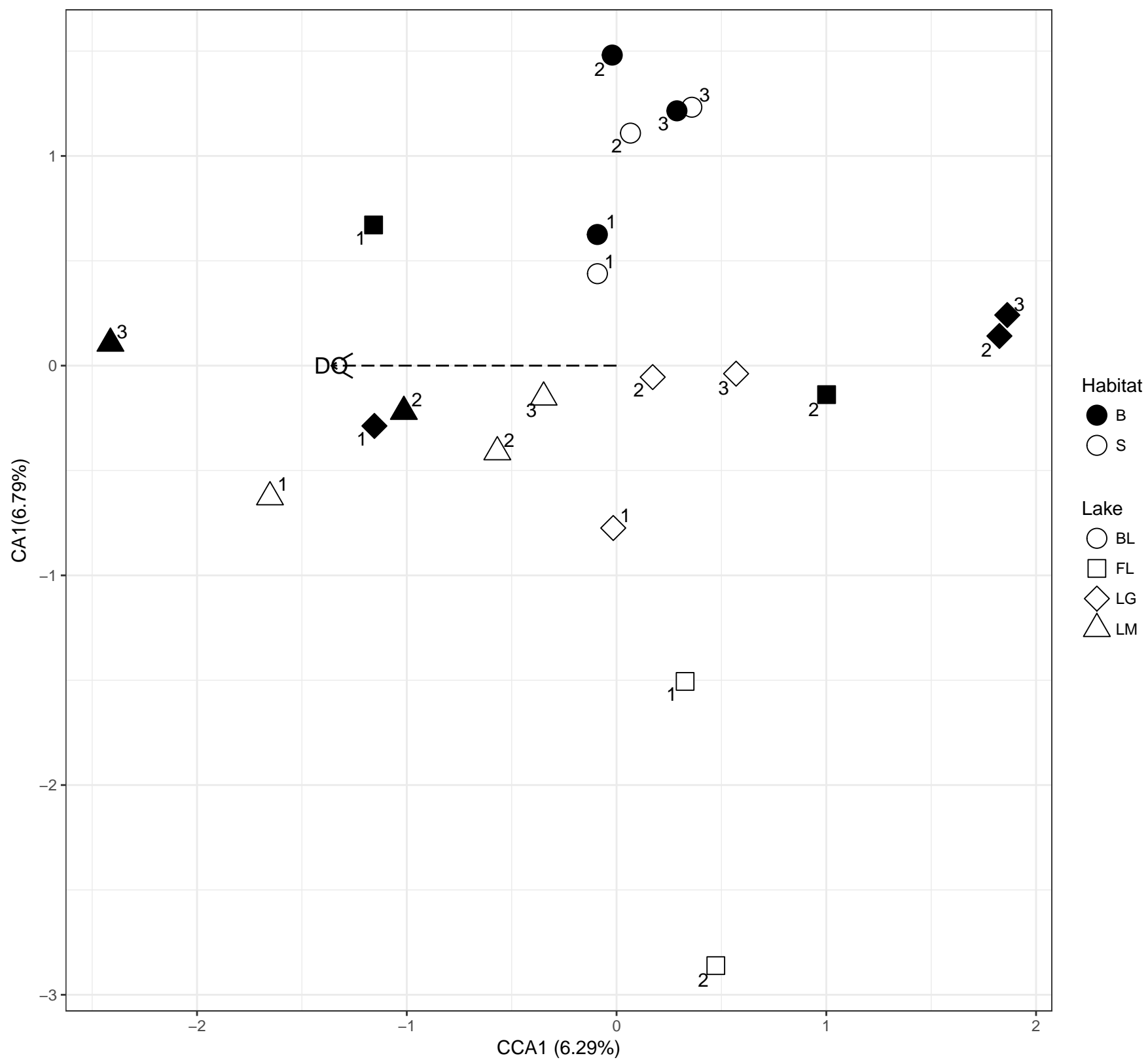

Supplement: Figure S2 — Partial Canonical Correspondence Analysis (pCCA) of microbial communities across lakes with oxygen as the constraining variable while controlling for variability caused by temperature and pH. Circles , Barney’s Lake, squares , Fox Lake, diamonds = Lake Geneserath, triangles , Lake Michigan. Open shapes correspond to surface-water samples, while filled shapes correspond to bottom-water samples. Numbers associated with points correspond to time point of sampling. Vectors correspond to environmental variables used to constrain variability in CCA. DO, dissolved oxygen. Percentages associated with axes correspond to percent total variability explained. [file peerj-05-3937-s002.pdf]
